# Real-world effectiveness of fremanezumab in patients with migraine switching from another mAb targeting the CGRP pathway: a subgroup analysis of the Finesse Study

**DOI:** 10.1186/s10194-023-01593-2

**Published:** 2023-05-23

**Authors:** Andreas Straube, Gregor Broessner, Charly Gaul, Xenia Hamann, Joachim Hipp, Torsten Kraya, Lars Neeb

**Affiliations:** 1 Department of Neurology, University Hospital, LMU Munich, Munich, Germany; 2 Department of Neurology, Innsbruck Medical University, Innsbruck, Austria; 3 Headache Center Frankfurt, Frankfurt, Germany; 4 Teva GmbH, Ulm, Germany; 5 Department of Neurology, Hospital Sankt Georg Leipzig gGmbH, Leipzig, Germany; 6 Department of Neurology, Headache Center Halle, University Hospital Halle, Halle (Saale), Germany; 7 Helios Global Health, Berlin, Germany; 8 Department of Neurology, Charité-Universitätsmedizin Berlin, Berlin, Germany

**Keywords:** Fremanezumab, Calcitonin gene-related peptide, Migraine, Non-responder

## Abstract

**Background:**

Monoclonal antibodies targeting the CGRP pathway are effective and safe for prophylactic treatment of episodic (EM) and chronic migraine (CM). In case of treatment failure of a CGRP pathway targeting mAb, physician has to decide whether using another anti-CGRP pathway mAb is useful. This interim analysis of Finesse Study evaluates effectiveness of the anti-CGRP mAb fremanezumab in patients with a history of other prior anti-CGRP pathway mAb treatments (switch patients).

**Methods:**

Finesse, a non-interventional, prospective, multicentre, two-country (Germany-Austria) study observing migraine patients receiving fremanezumab in clinical routine. This subgroup analysis presents data on documented effectiveness over 3 months after the first dose of fremanezumab in switch patients. Effectiveness was evaluated based on reduction in average number of migraine days per month (MMDs), MIDAS and HIT-6 scores changes as well as in number of monthly days with acute migraine medication use.

**Results:**

One hundred fifty-three out of 867 patients with a history of anti-CGRP pathway mAb treatment prior to initiation of fremanezumab were analysed. Switch to fremanezumab led to ≥ 50% MMD reduction in 42.8% of migraine patients, with higher response rate in EM (48.0%) than in CM patients (36.5%). A ≥ 30% MMD reduction was achieved by 58.7% in CM patients. After three months, monthly number of migraine days decreased by 6.4 ± 5.87 (baseline: 13.6 ± 6.5; *p* < 0.0001) in all patients, 5.2 ± 4.04 in EM and 7.7 ± 7.45 in CM patients. MIDAS scores decreased from 73.3 ± 56.8 (baseline) to 50.3 ± 52.9 (after 3 months; *p* = 0.0014), HIT-6 scores decreased from 65.9 ± 5.0 to 60.9 ± 7.2 (*p* < 0.0001). Concomitant use of acute migraine medication had decreased from 9.7 ± 4.98 (baseline) to 4.9 ± 3.66 (3 months) (*p* < 0.0001).

**Conclusions:**

Our results show that about 42.8% of anti-CGRP pathway mAb-non-responder benefit from switching to fremanezumab. These results suggest that switching to fremanezumab may be a promising option for patients experiencing poor tolerability or inadequate efficacy with prior other anti-CGRP pathway mAb use.

**Trial registration:**

Finesse Study is registered on the European Network of Centres for Pharmacoepidemiology and Pharmacovigilance (EUPAS44606).

**Graphical Abstract:**

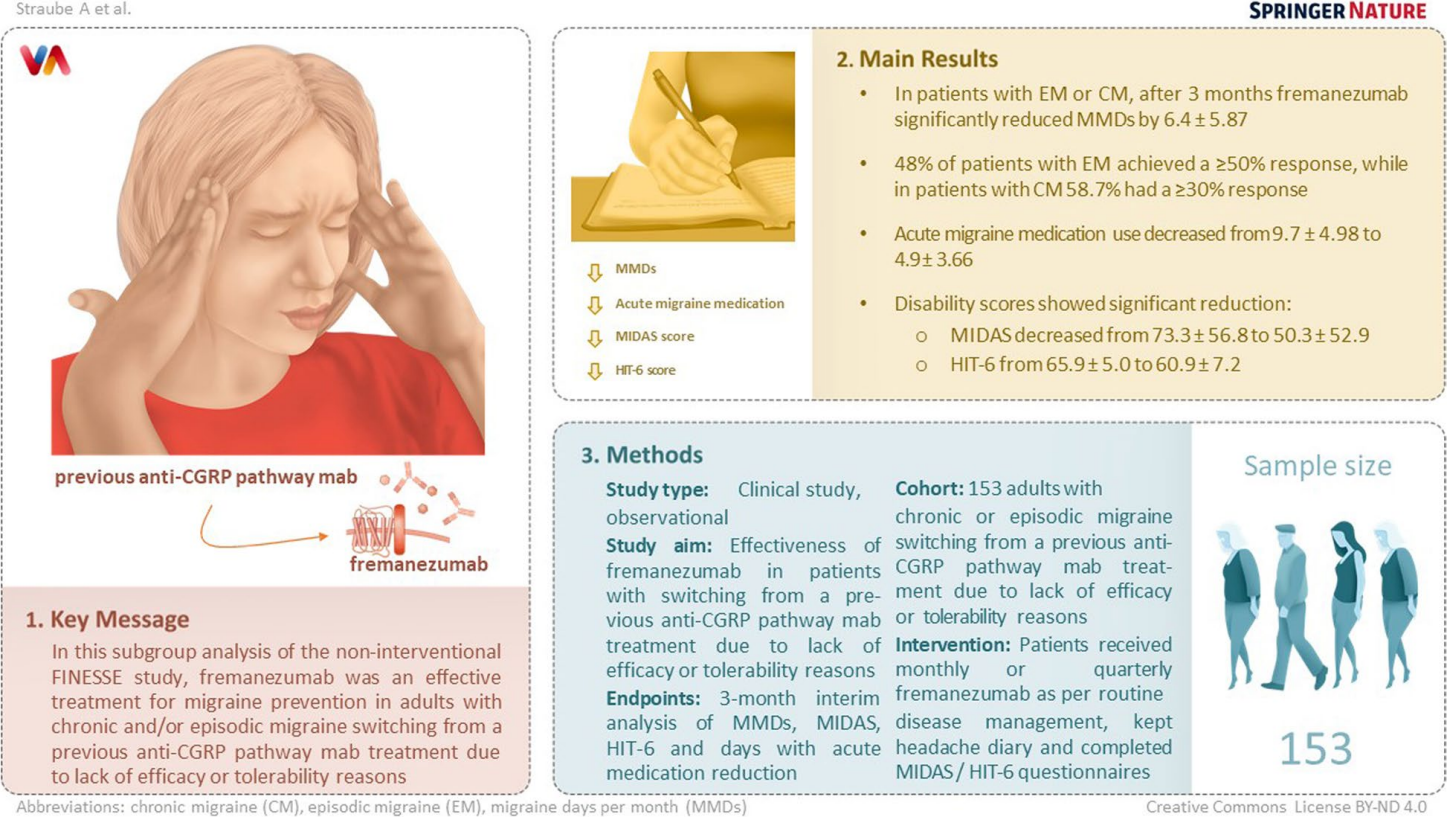

**Supplementary Information:**

The online version contains supplementary material available at 10.1186/s10194-023-01593-2.

## Background

Migraine is the most prevalent neurological disorder characterized by recurrent headache attacks that are moderate to severe and last in adults 4 to 72 h; associated symptoms may include nausea, vomiting, and sensitivity to light, sound, or smell [1, 2]. Migraine ranks among the world's most disabling medical illnesses, causing substantial economic and societal effect [3]. The discovery of the vasoactive neuropeptide calcitonin gene–related peptide (CGRP) [4] and the extensive research regarding the role of CGRP in migraine pathophysiology [5, 6] was a tipping point in the development of treatments targeting the CGRP pathway. CGRP is expressed in the trigeminal ganglion and is increased in the cranial venous outflow during acute migraine attacks. Specific antibodies either targeting the CGRP ligand (galcanezumab [7], fremanezumab [8], eptinezumab [9]) or the receptor (erenumab [10]) were developed and approved by the Food and Drug Administration (FDA) and European Medicines Agency (EMA). The diverse mechanisms of action of CGRP receptor versus ligand monoclonal antibodies may differentially affect efficacy, safety, and/or tolerability in migraine patients. The anti-CGRP pathway mAbs (receptor targeting or ligand binding mAb) are currently approved for the prophylaxis of migraine in adults who have at least 4 migraine days per month. Concerning the published results of the phase 3 RCT studies all four anti-CGRP pathway mAbs generally exhibit at least comparable reduction in MMDs equivalent to conventional migraine prevention drugs, and are generally superior with regard to safety, tolerability and low administration frequency in recent meta-analyses [11–13].

Treatment failure is not unheard for anti-CGRP pathway mAbs: a recently published real-life study included 864 patients treated with anti-CGRP pathway mAbs (erenumab 639 pts; galcanezumab; 173 pts; fremanezumab 55 pts) for ≥ 24 weeks. At week 24, 38.6% of CM patients and 35.1% of high-frequency episodic migraine (HFEM) patients did not achieve a ≥ 50% response rate [14]. Due to the diverse mechanisms of action of CGRP receptor versus ligand monoclonal antibodies, the question arises if a switch to other class of anti-CGRP pathway mAb is a reasonable strategy after the first class of anti-CGRP pathway mAb treatment failed. The non-interventional Finesse study aims to evaluate the overall effectiveness and tolerability of fremanezumab administered in adult patients with CM or EM in real-world clinical practice. This analysis of the prespecified subgroup presents effectiveness data of fremanezumab in patients with a history of prior treatment with another anti-CGRP pathway mAb. Reasons to switch to fremanezumab treatment are in most cases lack of efficacy, less often tolerability or other, not further specified reasons. Hereinafter referred to as failed treatment.

## Methods

### Study design

Finesse is an ongoing, 49-month, multicentre, two-country (Germany, Austria), prospective, non-interventional, observational study to describe outcomes of fremanezumab treatment in real-world clinical practice. Primary endpoint is the proportion of patients with a reduction in MMDs by ≥ 50%. Secondary objectives include measures of effectiveness of fremanezumab, such as changes in monthly average number of migraine days, disability scores (MIDAS, HIT-6 [15, 16]), use of concomitant acute migraine medication and patient adherence to and persistence with fremanezumab treatment. The patient follow-up is 24 months and the latest data analysis included in this paper was performed on August 12^th^ 2022.

All patients aged at least 18 years, with EM or CM – according to the criteria of the International Classification of Headache Disorders, 3^rd^ edition [17]—and completed headache diary for at least 21 days in the 28 days before fremanezumab initiation could be enrolled. The diary records, among other variables, MMDs and acute migraine medication use. Non-migraine headache and migraine-related disability was measured with the Migraine Disability Assessment (MIDAS) and the six-item Headache Impact test (HIT-6) as per local clinical routine. Patients were treated with fremanezumab monthly 225 mg or quarterly 675 mg as a treatment decision of their physician according to the SmPC with the first dose administered within three months of enrolment. As per observational plan, up to 30% of the enrolled patients could have previously been treated with other anti-CGRP pathway medications.

All patients provided written informed consent. The study was approved by the Ethical Committee of the Ludwig-Maximilian-University Munich and registered on the European Network of Centres for Pharmacoepidemiology and Pharmacovigilance (EUPAS44606).

### Statistical analysis

This publication presents results of the second interim analysis (IA), focusing on the three-month follow-up in patients who switched from another anti-CGRP pathway mAb to fremanezumab. The endpoint of this subgroup analysis is the percentage of patients with a ≥ 50% reduction in the monthly average number of migraine days during the three months after the first dose of fremanezumab. Further endpoints of this analysis include percentage of patients achieving ≥ 30% reduction in the monthly average number of migraine days during the three months after the first dose of fremanezumab, monthly number of migraine days, migraine severity assessed by MIDAS and HIT-6 scores, and use of acute migraine medication at baseline and month 3.

The analysis is primarily based on descriptive statistics. Categorical data were summarized by absolute number and percentage, metric data by arithmetic mean (µ), standard deviation (SD), quartiles, minimum, maximum, and sample size; here, µ ± SD are shown if not indicated otherwise. Wilcoxon test for patient reported outcomes (PROs) has been used. All statistical test results were assessed at a significance level of *p* ≤ 0.05.

## Results

**Fig. 1:**
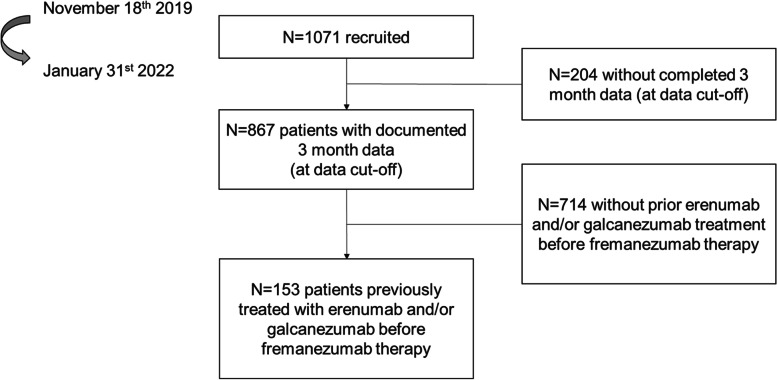
Flow chart of patients included

At all study centres, 1071 patients diagnosed with migraine were recruited starting November 18^th^, 2019 until January 31^st^ 2022. Out of 867 patients with completed three-month data at data cut-off (August 12^th^ 2022), 153 patients had received an anti-CGRP pathway mAb prior to fremanezumab treatment (Fig. 1).

### Demography and baseline characteristics

The baseline characteristics of the patients, who switched to fremanezumab (*n* = 153) were comparable to patients who received an anti-CGRP pathway mAb for the first time (*n* = 714), with the exception of age (45.0 ± 12.5 versus 48.0 ± 11.7 *p* = 0.0045), psychiatric disorders (39.5%) versus 55.6% *p* = 0.0003) and other neurological conditions (13.6% versus 20.3% *p* = 0.0438) (see Table 1).

**Table 1:** Demographic and selected baseline features, itemized by switch status. Categorical data are shown by N (%), metric data by µ ± SD. Percentages may not sum up to 100% in case of missing data

**Parameter**	**Non-Switch** (*N* = 714)	**Missing data (N)**	**Switch** (*N* = 153)	**Missing data (N)**	***p*-value**
**Demography**					
Sex [Female]	640 (89.6%)	-	128 (83.7%)	-	0.0487
Age [years]	45.0 ± 12.5	-	48.0 ± 11.7	-	0.0045
Caucasian Race	672 (94.1%)	-	145 (94.8%)	-	0.8504
**Height, Weight and BMI**					
Height [cm]	168.7 ± 7.6	279	168.4 ± 8.2	85	0.7717
Weight [kg]	71.9 ± 15.5	279	70.5 ± 15.6	85	0.4820
BMI [kg/m^2^]	25.3 ± 5.0	279	24.8 ± 5.2	85	0.5490
**Lifestyle**					
Alcohol consumption		95		85	0.1459
No Alcohol	273 (38.2%)		73 (47.7%)		
Irregular Alcohol	342 (47.9%)		63 (41.2%)		
Regular Alcohol	4 (0.6%)		0 (0.0%)		
Smoking status		87		13	0.5747
Non-Smoker	497 (69.6%)		110 (71.9%)		
Former Smoker	54 (7.6%)		14 (9.2%)		
Smoker	76 (10.5%)		16 (10.5%)		
**Medical History**^**a**^					
None	176 (24.6%)	-	25 (16.3%)	-	0.0268
Heart Diseases	78 (10.9%)	-	23 (15.0%)	-	0.1646
Endocrine Diseases	115 (16.1%)	-	28 (18.3%)	-	0.5482
Skeletal muscle, connective tissue and bone disorders	127 (17.8%)	-	34 (22.2%)	-	0.2083
Other neurological conditions	97 (13.6%)	-	31 (20.3%)	-	0.0438
Psychiatric diseases	282 (39.5%)	-	85 (55.6%)	-	0.0003

^a^Only SOCs reported for > 10% of total patients are shown

The analysis included 153 migraine patients with a history of anti-CGRP pathway mAb treatment prior to initiation of fremanezumab. Most of the patients (83.7%) were female; 94.8% were of Caucasian ethnicity. Mean age was 48.0 ± 11.7 years, with most patients (33.3%) in the age class 45—< 55 years. Median BMI was 24.8 kg/m^2^ (24.8 ± 5.2 kg/m^2^); thus, approximately half of the patients were overweight. Patients were predominantly abstinent from alcohol (47.7%) or irregular drinkers (41.2%), and predominantly non-smokers (71.9%) (Table 1).

Majority of the patients (83.7%) reported any medical history other than migraine (Table 1).

Most of the patients suffered from EM (52.3%), whereby 71.3% of the patients with EM experienced ≥ 8 days of migraine headache at baseline (the 28 days before initiation of fremanezumab) and were therefore classified as HFEM. Number of migraine days per month prior to first fremanezumab injection was 13.6 ± 6.5 (Table 2).

**Table 2:** Disease characteristics at baseline. Categorical data are shown by N (%), metric data by µ ± SD. Percentages may not sum up to 100% in case of missing data

**Parameter**	**Switch** (*N* = 153)
**Migraine Classification**	
CM	73 (47.7%)
EM	80 (52.3%)
High Frequency EM^a^	57 (37.3%)
Low Frequency EM^a^	23 (15.0%)
**Number of migraine days in 28 days prior to first dose**	
Total	13.6 ± 6.5
CM	17.7 ± 6.2
EM	9.9 ± 4.1

^a^EM patients with ≥ 8 days during the 28 days baseline period were regarded as “high frequency”, < 8 days as “low frequency”

All patients (100%) had a history of previous preventive migraine therapies. Reported medications were clearly dominated by anticonvulsants (94.1% of patients), antidepressants (92.2%) and beta-blockers (86.3%). Treatment duration varied between different classes of preventive therapies (Table 3).

**Table 3:** Past preventive migraine medication. Patients are shown by N (%), treatment duration by µ ± SD [months]. Percentages may not sum up to 100% in case of missing data

**Medication Class**	**Switch** (*N* = 153)	
	Patients	Treatment Duration (months)
Any prior preventive therapy	153 (100%)	N.A
Beta-blockers	132 (86.3%)	13.5 ± 23.3
Calcium Channel Blockers	88 (57.5%)	4.5 ± 7.1
Anticonvulsants	144 (94.1%)	9.4 ± 16.2
Antidepressants	141 (92.2%)	10.6 ± 17.6
Onabotulinumtoxin A	83 (54.2%)	10.6 ± 14.2
Any anti-CGRP pathway mAb	153 (100%)	N.A
Erenumab 70 mg	93 (60.8%)	6.4 ± 6.7
Erenumab 140 mg	110 (71.9%)	8.5 ± 7.1
Any erenumab	145 (94.8%)	10.6 ± 8.8
Galcanezumab	16 (10.5%)	7.4 ± 3.6

Medication history included combinations of different classes of medications. In switch patients, three (21.6%) or four (26.8%) medication classes prior to fremanezumab treatment including onabotulinumtoxin A were dominant, with a somewhat lower percentage of three (19.0%) or four (19.0%) classes without onabotulinumtoxin A.

Previous anti-CGRP pathway mAbs included galcanezumab in 16 (10.5%) of the switch patients and erenumab in 145 patients (94.8%; 70 mg in 60.8%, and 140 mg in 71.9%; an individual patient could be treated with both dosage). Eight of these switch patients had failed both mAbs (erenumab and galcanezumab) prior to initiation of fremanezumab. Treatment duration varied widely for anti-CGRP pathway mAbs as well, but was at maximum one year for galcanezumab, four years for erenumab 70 mg and three years for erenumab 140 mg. Previous anti-CGRP pathway mAbs had been more commonly discontinued due to lack of efficacy (galcanezumab: 62.5%, erenumab 70 mg: 87.1%, erenumab 140 mg: 85.5%) than for tolerability concerns (18.8%, 11.8% and 4.5%, respectively) or other, not further specified, reasons (18.8%, 8.6% and 10.9%, respectively).

### Responder rates / reduction in monthly migraine days

**Fig. 2:**
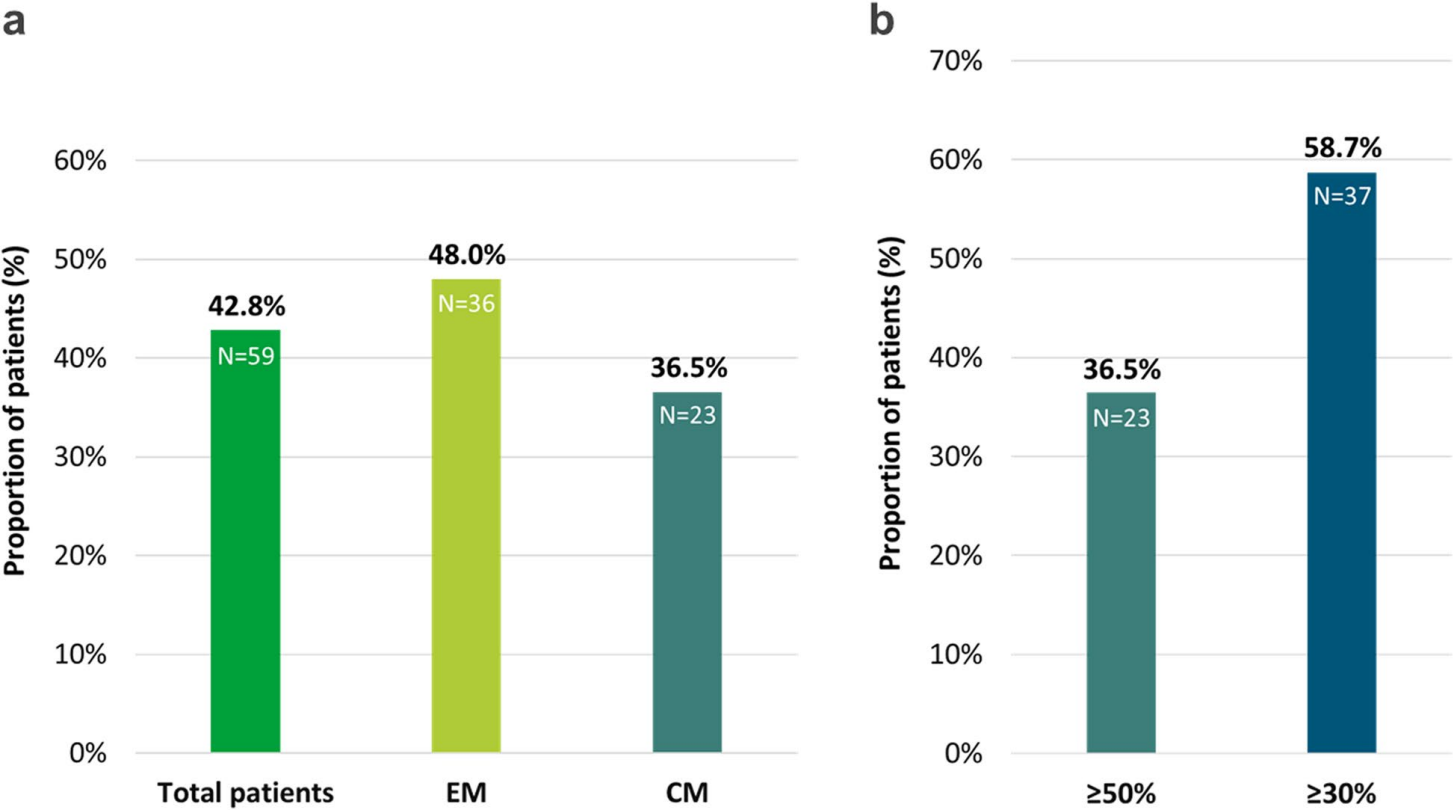
**a** Proportion of Patients with ≥ 50% Reduction in MMD over 3 months versus baseline **b** Proportion of CM-Patients with MMD reduction of ≥ 50% versus ≥ 30% over 3 months versus baseline

After three months, 42.8% of the patients achieved a 50% reduction in the MMDs (*n* = 138, missing data are excluded from the analysis (*n* = 15)), with a higher response rate in EM patients (48.0%) than in patients with CM (36.5%). Overall, 37 CM patients (58.7%) achieved a ≥ 30% response (Fig. 2a, b). An additional file shows response rate in more detail (see Table 5 supplementary file) In the subgroup of severely affected chronic migraine patients (≥ 24MMD) 8 out of 15 patients responded (≥ 50%). Two out of the eight patients (25.0%) who had previously failed to both anti-CGRP pathway mAbs responded (≥ 50%) to the fremanezumab therapy. Two patients who switched from galcanezumab showed a ≥ 50% response (2/8, 25%).

MMDs decreased from 13.6 ± 6.5 at baseline to 7.2 ± 5.5 at month 3 (*p* < 0.0001). The reduction was greater in CM patients (-7.7 ± 7.45; *p* < 0.0001) than in EM patients (-5.2 ± 4.0; *p* < 0.0001). In patients with at least 24 MMDs at baseline (*n* = 15, mean 26.2 ± 2.0) a reduction to 14.6 ± 8.9 MMDs was observed. Eight of these patients (53.3%) showed a ≥ 50% responder rate.

### Migraine disability scores

**Fig. 3:**
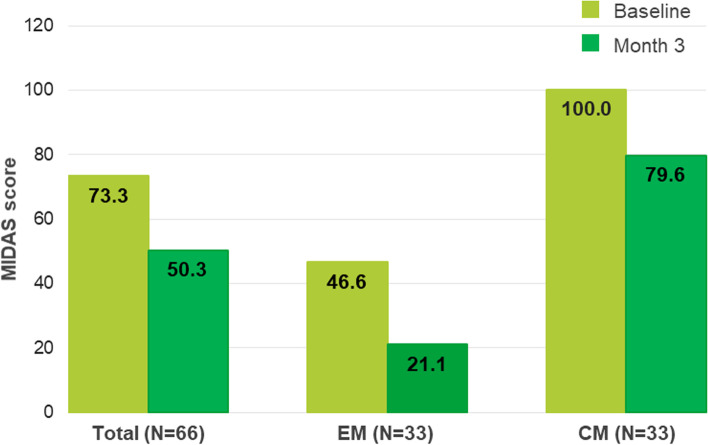
MIDAS score at baseline and at 3 months per migraine type

MIDAS scores decreased significantly from 73.3 ± 56.8 to 50.3 ± 52.9 at month 3 (*p* = 0.0014) in the total population, in EM patients from 46.6 ± 33.5 to 21.1 ± 17.4 (*p* = 0.0009) and in CM patients from 100.0 ± 62.9 to 79.6 ± 60.1 (*p* = 0.1225) respectively (Fig. 3). The MIDAS is categorized into four disability grades: grade 1 (MIDAS score 0–5) little or no disability; grade 2 (6–10) mild disability; grade 3 (11–20) moderate disability; grade 4 (≥ 21) severe disability [15]. MIDAS grade improved in 19 patients (28.8%), remained unchanged in 45 patients (68.2%) and worsened in 2 patients (3%).

**Fig. 4:**
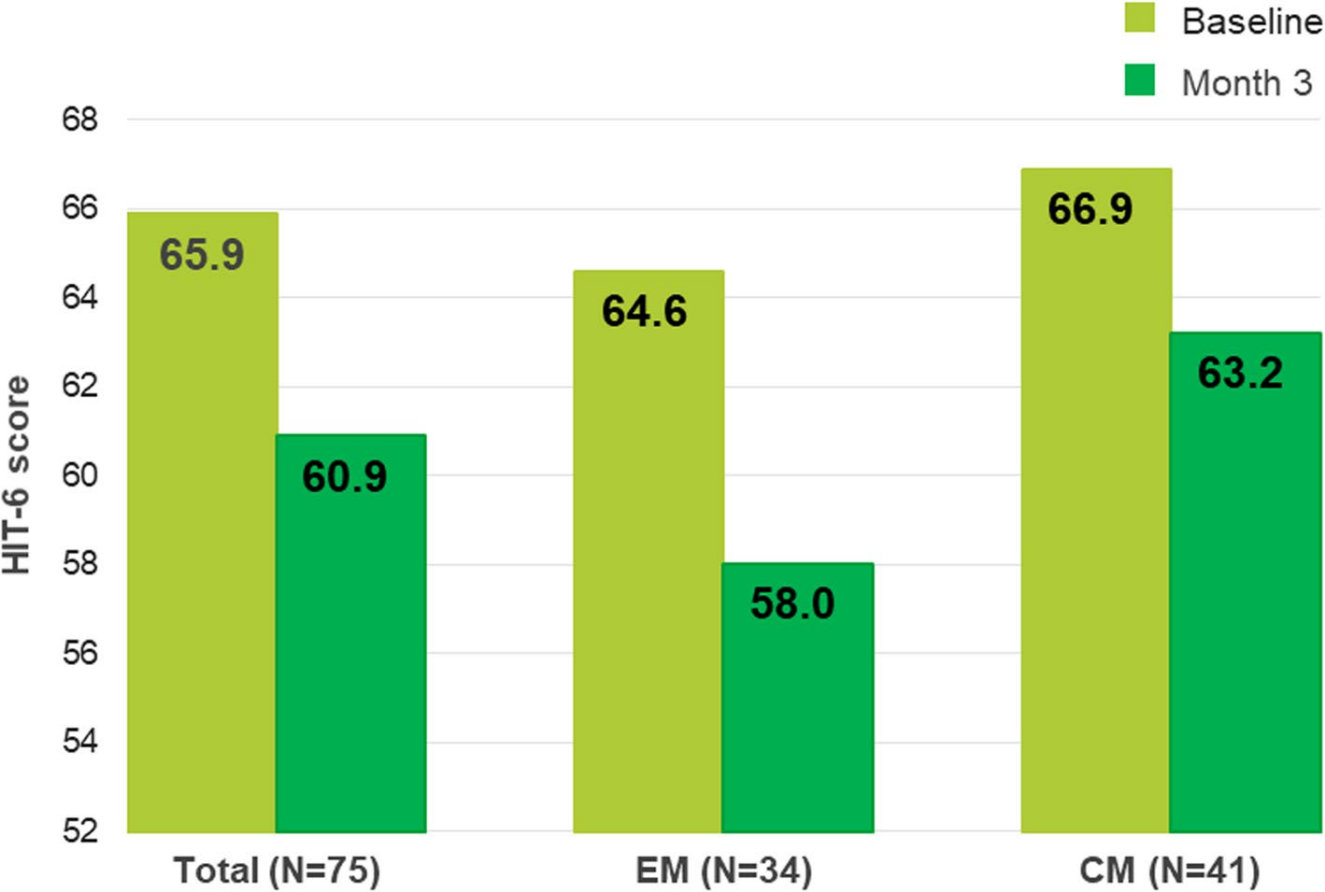
HIT-6 score reduction at 3 months per migraine type

In switch patients, HIT-6 scores decreased significantly from 65.9 ± 5.0 to 60.9 ± 7.2 at month 3 (*p* < 0.0001) in the total population, in EM patients from 64.6 ± 5.1 to 58.0 ± 7.7; *p* = 0.0004) and in CM patients from 66.9 ± 4.9 to 63.2 ± 6.0; *p* = 0.0052 respectively (Fig. 4).

A clinically relevant improvement of the HIT-6 score reduction of at least 5 points was found in 50.7% of the patients, 64.7% EM patients and 39% of the CM patients (Table 4).

**Table 4:** Patients with a HIT-6 reduction of ≥ 5 points from baseline to month 3 (missings excluded)

	**Switch patients with ≥ 5 points reduction**
**Total** (*N* = 75)	38 (50.7%)
**EM** (*N* = 34)	22 (64.7%)
**CM** (*N* = 41)	16 (39.0%)

### Acute migraine medication use

**Fig. 5:**
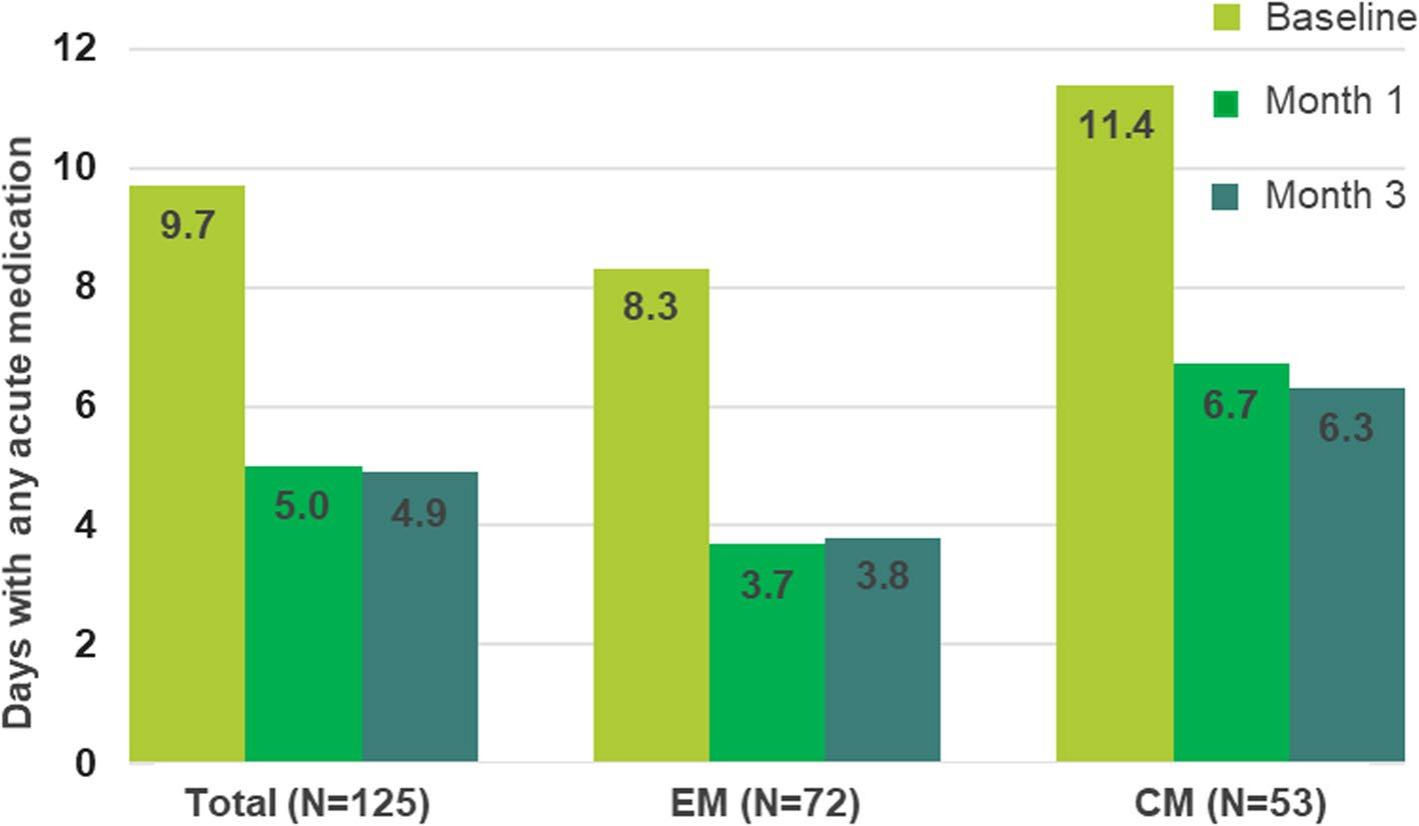
Monthly average number of days of any acute migraine medication use at baseline, month 1 and month 3

At baseline, acute migraine medication was used on 9.7 ± 4.98 days per month in all patients: after one month of treatment, only on 5.0 ± 4.37 days. In the EM subgroup, acute migraine medication use was consistently lower throughout the reporting period (8.3 ± 3.44 to 3.7 ± 2.75 days) than in CM (11.4 ± 6.11 to 6.7 ± 5.50 days). By month 3, acute migraine medication use had decreased to 4.9 ± 3.66 days (*p* < 0.0001) in the overall population. In EM and CM patients use of acute migraine medication decreased to 3.8 ± 3.09 days, and to 6.3 ± 3.91 days, respectively (*p* < 0.0001) (Fig. 5).

## Discussion

### Key results

This subgroup analysis explored the three month effectiveness of fremanezumab in this difficult to treat population, who failed at least one previous anti-CGRP pathway mAb treatment and other non- mAb preventives. Overall, at month three MMDs, days with acute medication usage as well as disability scores (MIDAS, HIT-6) improved.

Four anti-CGRP pathway mAbs are now authorized by the European Medical Agency for the prophylaxis of migraine in adults who have at least four migraine days per month. However, the use of anti-CGRP pathway mAbs is restricted by different reimbursement conditions. During the recruitment period of Finesse Study, a prerequisite for reimbursement in Germany for adult patients was: adult patients who do not respond to, inability to tolerate, discontinuation due to side effects, and/or contraindication from treatment with at least four (EM) or five (CM) migraine preventives. In Austria, three migraine prophylaxis treatment failure are necessary prior to treatment with an anti-CGRP pathway mAb (whereby failed is defined as no improvement / failure: no relevant clinical response/not tolerated due to side effects or contraindicated) to be reimbursed.

The diverse mechanisms of action of CGRP receptor versus ligand monoclonal antibodies may differentially affect efficacy, safety, and/or tolerability in migraine patients. Patients who did not respond to one anti-CGRP pathway mAb class may benefit from a switch to the other class [18].

Fremanezumab antagonizes CGRP-induced cAMP signalling at the CGRP receptor, by targeting the peptide. In contrast, erenumab antagonizes CGRP, adrenomedullin and intermedin cAMP signalling at the canocical human CGRP receptor. Furthermore, erenumab but not fremanezumab, binds to the canonical human CGRP receptor and internalises [19]. Most authors discuss a peripheral effect (outside the blood brain barrier) of the mAbs. Recently, functional magnetic resonance imaging studies suggest possible different central responses to the ligand antibody galcanezumab and to the anti-CGRP receptor antibody, erenumab. Galcanezumab led to a decrease in activity in the hypothalamic area, left thalamus and in pontine region, whereas erenumab specifically decreased activation in the operculum, insula, thalamus and cerebellum [20, 21].

No interventional clinical trial has evaluated the effect of switching to another anti-CGRP pathway mAb in migraine patients who have not previously responded to an anti-CGRP pathway mAb so far. However, limited real-world data are available ranging from the first case report [22], mono-centric case series [23–25] or retrospective cohort studies [26, 27].

This subgroup analysis shows data from a prospective, multicentre (> 100), two country non-interventional study. The primary endpoint of this subgroup analysis evaluated a ≥ 50% responder rate over 3 months, additionally a ≥ 30% responder rate in CM, which is considered clinically meaningful [28, 29].

Single center, retrospective case series in patients with prior exposure to another CGRP pathway-targeted mAb showed ≥ 50% responder rates over / at 3 months from 20%-45.5% [25, 27]. A published non-peer reviewed retrospective case series showed a similar percentage of responders irrespective of the fact that patients switched from a receptor antibody to a ligand antibody or if they switched within the ligand antibody class [24]. The 42.8% response rate of MMDs reduction in our study was in line with results previously reported in a retrospective panel-based chart review study [27].

It remains unclear why some patients respond to treatment with an anti-CGRP pathway mAb and some do not. Predicting a clinical response to migraine preventives is in general not possible, neither based on clinical variables nor on molecular markers [14]. However, a recent prospective multicentre real-life study showed a higher likelihood of ≥ 50% response to anti-CGRP pathway monoclonal antibodies over 24 weeks in HFEM patients with unilateral pain and unilateral cranial autonomic symptoms (OR: 4.23; 95%CI: 1.57–11.4; *p* = 0.004) while in CM patients the response was negatively associated with obesity (OR: 0.21, 95%CI: 0.07–0.64; *p* = 0.006) [14]. Concerning a positive response to switching to another anti-CGRP pathway mAb the retrospective case series published by Overeem et al. showed that none of the 9 patients with chronic daily headache (no headache free day) did respond to the switch whereby 50% of the patients with non-daily chronic headaches improved by at least 30% [26]. A retrospective study in anti-CGRP pathway mAb naϊve migraine patients with chronic daily headaches (24 MMDs in median) showed a low 50% responder rate in monthly headache days (MHD) (13%) and a higher responder rate in MMDs (25%) [30]. In our cohort we did not capture monthly headache days. In the small subgroup of severely affected chronic migraine patients (≥ 24 MMDs, mean 26.2 ± 2.0) we observed a ≥ 50% responder rate in 8 patients (8/15 patients, 53.3%).

In the small sample of patients included in FINESSE, previously treated with the ligand antibody galcanezumab a ≥ 50% responder rate was observed in 25% (2/8) of the patients. In patients who failed both, erenumab and galcanezumab treatment also a 25% responder rate was achieved. But the groups are too small to draw further conclusions.

The herein presented analysis has several strengths and limitations. This prospective study analyses the effects of a switch to fremanezumab in patients who did not respond to or discontinued a previous treatment with an anti-CGRP pathway mAb due to tolerability concerns. The main limitation lies in the non-interventional study design, entailing gaps in recording clinical data over time and only data captured in the centre specific routine care are recorded. This effect is predominately seen in the capture of patient reported outcomes (PROs: MIDAS, HIT-6). About half of the patients (*n* = 75) has documented PRO values at baseline and at 3 months. Further, insufficient response and treatment failure was assessed according clinical routine and not defined by a threshold e.g. less than ≤ 30% reduction of MMDs before switching to fremanezumab.

Since erenumab was approved by EMA earlier than the ligand anti-CGRP antibodies, more patients were pretreated with that antibody. A fact that explains why most of the included patients switched from erenumab (*n* = 137), followed by galcanezumab (*n* = 8) and eight patients had been previously treated with both mAbs. Therefore, the results of this analysis are more generalizable to a switch from the receptor anti-CGRP mAb, and to a lesser extent to a switch from a ligand anti-CGRP mAb. The majority of patients (> 80%) discontinued their previous treatment with an anti-CGRP pathway mAb due to non-satisfactory response. Strength of the study is the large number of patients and their recruitment from an extended geographical area (Germany and Austria) in a large number of centres. The broad inclusion criteria allowed the inclusion of all age classes (≥ 18 years) and patients with comorbidities like psychiatric disorders. These results therefore seem to represent the real-world situation in the participating countries better than the phase 3 studies.

Consistent with previous case series and studies, this subgroup analysis of FINESSE found that switch patients were still likely to benefit from fremanezumab treatment. Even in a hard to treat patient population with prior preventive treatments and ≥ 1 anti-CGRP pathway mAb treatment failure, high migraine frequency (13.6 days/month), frequent CM (47%) and high disability (MIDAS 73.3), switching to fremanezumab could be a reliable option for those patients who failed a previous anti-CGRP pathway mAb.

## Conclusions

Our analysis in 153 patients shows that 42.8% of the patients, who failed a previous anti-CGRP pathway mAb therapy demonstrated a ≥50% reduction of MMDs after switching to fremanezumab. Clinically relevant improvement in patient reported outcome measures (MIDAS, HIT-6) as well as reduction in the number of monthly average days of concomitant acute migraine medication intake was achieved. These data provide prospective real-world evidence that treatment with fremanezumab may results in clinical benefit in patients who previously failed other anti-CGRP pathway mAb treatment.

### Trial registration

FINESSE study is registered on the European Network of Centres for Pharmacoepidemiology and Pharmacovigilance (EUPAS44606). Retrospectively registered on December 8^th^ 2021. Previously registered at Paul-Ehrlich-Institut (Federal Institute for Vaccines and Biomedicines) on November 11th 2019.

## Supplementary Information

**Additional file 1.**

## Data Availability

The datasets generated during and analysed in the frame of this study are available from the corresponding author on reasonable request.

## Abbreviations

BMI: Body Mass Index
CGRP: Calcitonin Gene-Related Peptide
CM: Chronic Migraine
EM: Episodic Migraine
HFEM: High Frequency Episodic Migraine
HIT-6: Headache Impact Test 6
IA: Interim Analysis
mAb: Monoclonal Antibody
MMDs: Migraine Days per Month
MHD: Headache Days per Month
MIDAS: Migraine Disability Assessment
N.A.: Not Applicable
PRO: Patient Reported Outcome
SD: Standard Deviation
SmPC: Summary of Product Characteristics
SOC: System Organ Class
Switch Patient: Patient receiving fremanezumab in the frame of clinical routine who has a history of prior treatment with any other anti-CGRP pathway mAb
µ: Arithmetic Mean
95% CI: 95% Confidence Interval
