# Supplementary material for: Real-world effectiveness of fremanezumab in patients with migraine switching from another mAb targeting the CGRP pathway: a subgroup analysis of the Finesse Study
Source: J Headache Pain. 2023 May 23;24(1):59. doi: 10.1186/s10194-023-01593-2 (PMC10207758; doi:10.1186/s10194-023-01593-2)
Supplement: Supplementary file 1 — Additional file 1. [file 10194_2023_1593_MOESM1_ESM.docx]

**Table 5: Patients achieving 30%, 50% and 75% reduction in monthly average number of migraine days during three months after first dose of fremanezumab shown by N (%), missings excluded**

| **Disease Characteristics** | | **Reduction in monthly average number of migraine days during 3 months** | | |
| --- | --- | --- | --- | --- |
| Switch Status | Migraine Type | By ≥30% | By ≥50% | By ≥75% |
| **Switch** N = 153 | Total | 95 (68.8%) | 59 (42.8%) | 19 (13.8%) |
|  | EM | 58 (77.3%) | 36 (48.0%) | 12 (16.0%) |
|  | CM | 37 (58.7%) | 23 (36.5%) | 7 (11.1%) |
